# Trans-oral robotic surgery for a Ewing's sarcoma of tongue in a pediatric patient: a case report^☆^

**DOI:** 10.1016/j.bjorl.2017.04.001

**Published:** 2017-05-02

**Authors:** Frank Rikki Canevari, Filippo Montevecchi, Stefania Galla, Raffaele Sorrentino, Claudio Vicini, Federico Sireci

**Affiliations:** a S.S. Antonio Biagio e Cesare Arrigo Hospital, Otorinolaryngology Section, Alessandria, Italy; b L. Pierantoni Hospital, G.B. Morgagni, ENT and Oral Surgery Unit, Forlì, Italy; c P. Giaccone Hospital, Department of Experimental Biomedicine and Clinical Neurosciences (BioNeC), Otorhinolaryngology Section, Palermo, Italy

## Introduction

The Ewing's sarcoma Family of Tumors (EFT) includes classic Ewing's Sarcoma (ES) of bone, Extraskeletal Ewing's Sarcoma (EES) and malignant peripheral primitive Neuroectodermal Tumor (pNET) of bone and soft tissue.

ES is an aggressive tumor with a high incidence of local recurrence and distant metastasis, which is more common in males respect to females, particularly in the first 2–3 decades of life.^1^ The skeletal form is more common and typically occurs in the long bones of the extremities. The extra skeletal form occurs in the soft tissues of the lower extremities, paravertebral tissues, chest wall, retroperitoneum and rarely in the head and neck region in about 1–4% of cases.^2^ Involvement of the head and neck is usually identified in the nasal or oral cavities, sinuses or soft tissues of the neck.^3^ Primary Ewing's sarcoma of the base of tongue is exceedingly rare and we present the first case in literature in a 16 year-old male treated by Trans-Oral Robotic Surgery (TORS) and postoperative chemotherapy.^4^

## Case report

A 16-year-old male patient presented to our institute in June 2016 with a history of 6 months of odynophagia. Laringoscopy revealed a red mass located in the right part of base of tongue extending to homolateral glossoepiglottic vallecula with partial obstruction of airway. No enlarged lymph nodes were palpated.

**Figure 1:**
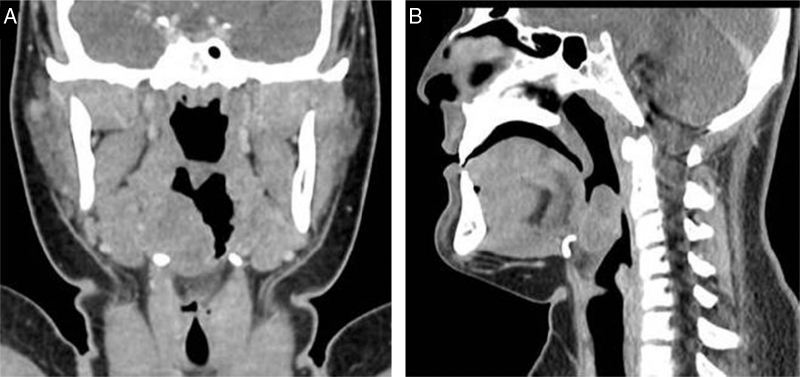
Coronal (A) and Sagittal (B) Computerized Tomography, with contrast media, showing a mass in the right part of tongue base. No neck lymphadenopathy were evidenced.

Total body contrast-enhanced Computed Tomographies (CT) scan revealed a 3–3.2 cm isointense mass located in the right part of tongue base extended to soft tissue with soft tissue surrounding and with a very poor contrast enhancement. No information of the nature of the mass was achieved by imaging. No neck lymphadenopathy and metastasis were evidenced (Fig. 1). Due to the young age of the patient, a biopsy under general anesthesia was performed using Trans-Oral Robotic Surgery (TORS) approach. A Crowe-Davis mouth gag was used to expose the tumor. The daVinci Surgical System (Intuitive Surgical Inc., Sunnyvale, CA) utilizing 8 mm 08 and 308 endoscopes alternately for visualization, a 5 mm Maryland forceps on the right arm and a 5 mm monopolar cautery with disposable spatula tip on the left arm of the daVinci Surgical System was used to perform the resection.

**Figure 2:**
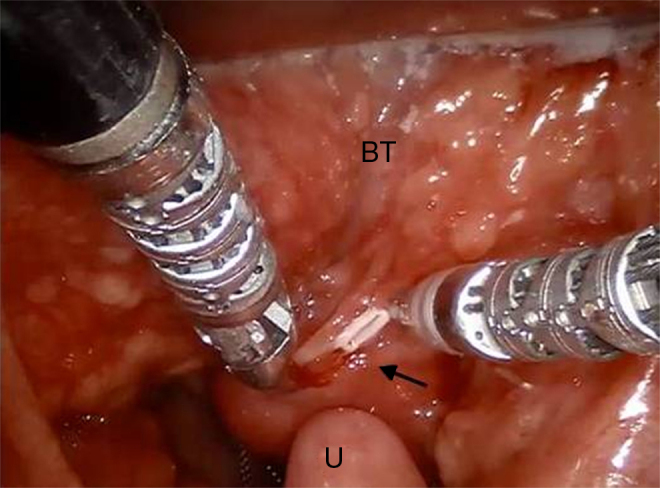
Intraoperative view of surgical field. Excellent one view exposure of tumor (arrow), dorsal and medial Base of Tongue (BT) and Uvula (U).

Intraoperative frozen histology sections was performed but not diriment and therefore anatomopathologist asked for a larger amount of tissue. We decided to remove completely the mass performing partial glossectomy (Fig. 2).

The margin of the resection, examinated with introperative histology, were free of any disease.

**Figure 3:**
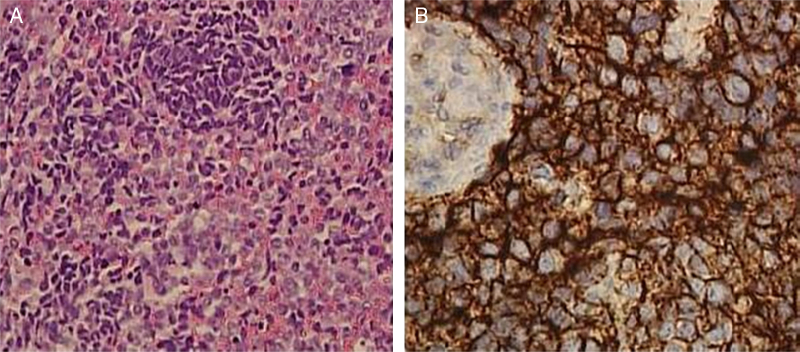
(A) The mass consisted of diffuse sheets of small round blue cells with scant cytoplasm and inconspicuous nucleoli (hematoxylin-eosin, original magnification 20×); (B) Immunohistochemistry for CD99 demonstrated diffuse, membranous staining of tumor cells (40×).

Histological examination revealed a Extraskeletal Ewing's Sarcoma (EES). The microscopic examination of the specimen revealed a population of epithelioid cells with round nuclei and scant cytoplasm, a high number of mitotic figures and focal necrosis. The tumor cells were immunohistochemically positive for CD99 and Bcl-2 and negative for leukocyte common antigen (CD45), S-100, cytokeratin AE1/AE3 and desmin. Furthermore, interphase Fluorescence In Situ Hybridization (FISH) revealed a t(22;11) translocation (Fig. 3).

The patient was discharged after 2 days and recovered after 15 days.

The patient began chemotherapy with ifosfamide, etoposide, vincristine, doxorubicin, cyclophosphamide ×4 cycles each. There has been complete response to therapy with no evidence of recurrence on CT imaging after 4 months.

## Discussion

Ewing Sarcoma (ES) was first described in 1921 by James Ewing. It's a highly malignant bone tumor that involves children and young adults. Currently these tumors are classified as ES Family of Tumors (EFT) and include Ewing Sarcoma (ES) Extraskeletal Ewing Sarcoma (EES) and Primitive Neuroectodermal Tumor (PNET), which shows more neural differentiation.

The imaging findings are nonspecific in EES, and can help only in localization, extension of the tumor and involvement of surrounding anatomical structures. The reported CT findings in ESS is mostly a mild heterogeneously enhancing mass. On MRI has an isointense signal in T1 if compared to muscle, a hyperintense signal in T2 weighted images and a low and heterogeneous enhancement.

Our case showed a mild contrast enhanced mass without bony structure, vascular structure and lymphonodes of the neck involvement. Imaging findings were not suggestive for any diagnosis so we decided to perform a biopsy in order to plan further treatment.

The diagnosis of EES is histological with a typical immunohistochemical pattern.

Histologically ESS consists of a monotonous population of small, round, hyperchromatic cells with scant cytoplasm. The malignant cells appear undifferentiated and frequently lack any clear morphologic features. Necrosis, Homer-Wright rosettes and a background with neurofibrillary material are variably present.^5^ Immunohistochemical staining of ESE is usually distinct and shows diffuse CD99 (MIC2), FLI-1 and vimentin positivity. Variable positive staining for cytokeratin, in about 20% of cases, and neural markers including Neuron Specific Enolase (NSE), synaptophysin and S-100 can be seen. Typically tumors with more differentiation toward pNET exhibit increased staining for neural markers. Perhaps most helpful is negative staining for CD45 (leukocyte common antigen), myogenin/MyoD1, Epithelial Membrane Antigen (EMA), chromogranin and HMB-45 which help to preclude important histologic mimickers from the differential including lymphoma, rhabdomyosarcoma, salivary gland tumors and nasopharyngeal carcinoma, olfactory neuroblastoma and melanoma respectively.

Confirmation of histologic findings can be achieved through molecular and cytogenetic studies. The characteristic chromosomal translocation t(11;22)(q24;12) is found in about 90% of ES, ESS and Pnet.^6^

EES are rare that and therefore guidelines regarding therapy and surgery are still discussed.

Complete surgical excision, when feasible, is regarded as the best modality of local control, given the higher risk of local recurrence when radiotherapy is used as the sole treatment of the primary tumor. Radiotherapy alone (in the range of 45–60 Gy) should be applied if complete surgical excision is impossible. Postoperative radiotherapy should be given in cases of inadequate surgical margins or in presence of metastases and therefore, in our case, it was not carried out. Combining surgery and chemotherapy, survival is ∼60% to 70% in localized tumor. All current trials employ 6–10 cycles of chemotherapy usually applied at 2 to 3 week intervals. Treatment duration is thus 10–12 months. Agents considered most active include doxorubicin, cyclophosphamide, ifosfamide, vincristine, dactinomycin, and etoposide.^7^

About surgical strategy, we decided to perform a Trans-Oral Robotic Surgery (TORS). TORS has been used with good results for benign pathologies such as the surgical treatment of sleep apnea (OSAS)^8^ but in the last years it has imposed itself in the treatment of early stage (T1–T2) for base of tongue neoplasms.

Over the past 10 years, there have been increasing reports of the use of primary radiation or combined chemotherapy and radiation for tongue base neoplasms. The key factor driving this movement away from primary surgery was the reported morbidity of such surgical procedures.

Cervical incisions and dissections with mandibulotomy or pharyngotomy were typically required to remove base of tongue neoplasms even in the early stages. These approaches left the patient with various levels of significant speech and swallowing dysfunction as well as cosmetic deformity. The introduction of endoscopic laser microsurgery for tongue base cancer has been reported by Steiner et al.^9^ Transoral CO_2_ laser pharyngeal surgery reintroduced primary surgery as a means of treating base of tongue cancer, but this technique is technically challenging, has a steep learning curve, and a limited operative field of view because it is performed through a laryngoscope. Furthermore, the transoral laser surgery requires cutting directly through the tumor to determine extent of resection. Nonetheless, the transoral approach did reveal that 92% of patients achieved swallowing without permanent gastrostomy tube.

We believe that TORS for tongue base lesions has significant advantages over both classical open tongue base surgery and laser microsurgery. With respect to open approaches, it is well known and widely reported that open surgery of the tongue base has obvious negative impact on both functional and cosmetic outcomes. TORS eliminates risk of fistula and infections and the need for mandibulotomy with a lip split or visor flap or transpharyngeal approaches that adversely affect chewing, swallowing and speech function, and cosmesis. In addition, we believe that TORS tongue base resections can be performed safely without tracheostomy, which is typically used for open approaches.

TORS offers several potential advantages over laser base of tongue surgery as well. Although the standard operating microscope used in laser procedures provides excellent direct visualization to an exposed area, it cannot view around corners or cannot be rotated along three-dimensional axes. The 0 or 30 degree angles and the maneuverability of the robotic endoscopes is a key issue in the ability to achieve en bloc resections of the tumors with negative margins like as in our case. Laser microsurgery is essentially one-handed surgery, whereas robotic surgery is two- or even four-handed (if the assistant is included), and tissue manipulation and retraction is comparable with open surgery. It is our impression that TORS offers a wider variety of technical options for hemostasis compared with transoral laser microsurgery. Hemostasis was easily managed in the live surgeries with either monopolar or bipolar cautery robotic instrumentation and the use of small-sized hemoclips.^10^

## Ethical approval

The patient has signed an informed consent allowing us to use his medical records.

## Conclusions

Extraskeletal Ewing's sarcoma are rare tumor but that can arise in the head and neck region. A combination of multiple diagnostic modalities, surgery and chemotherapy represent the correct treatment in these cases. In particular for the tumors of base of tongue, TORS offers a complete excision of tumor and therefore a best outcome.

## Conflicts of interest

The authors declare no conflicts of interest.
